# An Evaluation of Exposure to 18 Toxic and/or Essential Trace Elements Exposure in Maternal and Cord Plasma during Pregnancy at Advanced Maternal Age

**DOI:** 10.3390/ijerph192114485

**Published:** 2022-11-04

**Authors:** Tingfei Gu, Xiaoqian Jia, Huifeng Shi, Xiaoli Gong, Jinxi Ma, Zhihang Gan, Zhixin Yu, Zhiwen Li, Yuan Wei

**Affiliations:** 1 Department of Obstetrics and Gynecology, Peking University Third Hospital, Beijing 100191, China; 2 Health Science Center, Peking University, Beijing 100191, China; 3 Institute of Reproductive and Child Health, Peking University, Beijing 100191, China; 4 Key Laboratory of Reproductive Health, National Health Commission of the People’s Republic of China, Beijing 100191, China; 5 Department of Epidemiology and Biostatistics, School of Public Health, Peking University, Beijing 100191, China; 6 National Clinical Research Center for Obstetrics and Gynecology, Peking University Third Hospital, Beijing 100191, China; 7 National Center for Healthcare Quality Management in Obstetrics, Beijing 100191, China

**Keywords:** maternal plasma, umbilical cord plasma, essential trace elements, toxic elements, advanced maternal age

## Abstract

Pregnant women of advanced maternal age (AMA) are vulnerable to exposure to the surrounding environment. Assessment of trace elements in pregnant women living in specific areas is important for biomonitoring. However, exposure levels and variation patterns during pregnancy remains controversial and attracts extensive public concern. Therefore, we aimed to evaluate exposure of 18 toxic and/or essential trace elements in maternal plasma and in paired cord plasma during pregnancy at AMA. A total of 48 pregnant women of AMA were recruited in Peking University Third Hospital from 2018 to 2021. Eighteen elements found in maternal plasma during the 1st, 2nd, or 3rd trimester of pregnancy and paired cord plasma were measured by 7700x ICP-MS (Agilent Technologies, Palo Alto, CA, USA) and Elan DRC type II ICP-MS (The Perkin-Elmer Corporation, Waltham, MA USA). Concentrations of Pb, Se, Fe, Zn, and Mo all decreased during pregnancy, while Cu increased. Interestingly, concentrations of Rb decreased initially but then increased. Elements as Al, Co, Se, Cu, and Ni showed significantly lower levels in cord than in maternal plasma, while elements as Sr, Fe, Rb, Mn and Zn displayed significantly higher levels in cord than in maternal plasma. Moreover, positively- interacted clusters were found in Ni-Co-Cu-Al-Rb-Zn and Zn-Mn-Al-Pb in maternal blood. Similar positively-interacted clusters were found in Zn-Ni-Co, Zn-Ni-Fe, Mn-Al-Pb, Fe-Pb-Mn, Fe-Ni-Cu, and Rb-Cu-Sb-Fe-Mn in cord plasma. Furthermore, correlations between paired maternal and cord blood samples for As, Sr, and Mo were statistically significant, indicating that the fetus burden may reflect maternal exposure to some extent. Admittedly, levels of toxic and essential elements in our cohort study were comparatively lower than those in the scientific literature.

## 1. Introduction

Vulnerable to adverse effects of the surrounding environment, pregnant women and fetuses undergo a daily exposure to pollutants from several pathways. Previous studies have already reported the detrimental association of prenatal arsenic (As), cadmium (Cd), and lead (Pb) exposures with anthropometric parameters of newborns, such as birth weight, birth length, and head circumference [1,2,3]. The effects can extend to early childhood and may affect the later risk of diseases [4].

In contrast to these toxic elements, maternal trace element status is important for a healthy pregnancy. Insufficient supplies of essential micronutrients can lead to a state of biological competition between the mother and conceptus, which can be detrimental to the health status of both [5]. Deficiencies of specific antioxidant activities associated with the micronutrients selenium (Se), copper (Cu), zinc (Zn), and manganese (Mn) can result in poor pregnancy outcomes, including fetal growth restriction [6], preeclampsia [7], and the associated increased risk of diseases in adulthood, including cardiovascular disease and type 2 diabetes [8,9].

Previous studies have already reported some changes in element levels during pregnancy. Cu and Zn levels significantly increased in maternal blood at delivery compared to the 1st trimester of pregnancy, arising from mothers’ increased metabolic demand for these nutrients [10]. In addition, maternal blood Mn levels could increase during pregnancy because of increased intestinal absorption, and possibly increased binding capacity towards Hb to ensure metal sufficiency [11].

Placenta and umbilical cord blood play an essential role in the placental transfer of elements, which can be assessed by comparing their concentrations in maternal and cord blood [12]. Several toxicants are proven to totally or partially cross the placental barrier [13]. In particular, As, Cd, mercury (Hg), and Pb could extend the health risk to the fetus, even at low levels, through transplacental circulation.

Advanced maternal age (AMA) has historically been defined as ≥35 years at the time of delivery [14]. It should be stressed that since the implementation of the universal two-child policy, the percentage of AMA in China has rapidly increased [15], and AMA pregnancy is proven to be associated with higher risks of adverse perinatal outcomes [16].

However, to our knowledge, exposure to other toxic and essential trace elements during pregnancy has still been a largely under-explored domain, particularly with regard to AMA pregnancy. In order to fill this gap, our prospective study focuses on perinatal exposure to 18 elements, including 6 toxic (Aluminum (Al), As, Cd, Hg, Pb, antimony (Sb)) and 12 essential trace (Boron (B), Cobalt (Co), Chromium (Cr), Copper (Cu), Iron (Fe), Nickel (Ni), Manganese (Mn), Selenium (Se), Zinc (Zn), Rubidium (Rb), Strontium (Sr), Molybdenum (Mo)) elements. By studying the variation pattern of these elements in maternal plasma during pregnancy and in paired cord plasma, we aim to offer some suggestions on the management of AMA pregnancy to reduce adverse perinatal exposure if possible.

## 2. Materials and Methods

### 2.1. Study Design and Data Collection

This is a prospective cohort study using data and samples from the maternal cohort in Peking University Third Hospital. A total of 48 pregnant women were included in the cohort, using the following inclusion criteria: (1) women with singleton pregnancies; (2) women with complete measuring information in 1st, 2nd, 3rd trimester and cord plasma; (3) women with AMA (from ages 35 to 42). 

All patients were fully informed of the content and purpose of the study and gave written informed consent. All the procedures of this study were reviewed and approved by the Medical Science Research Ethics Committee of the Peking University Third Hospital (IRB00006761-2016145).

### 2.2. Sample Collection and Laboratory Analyses

Maternal whole blood (about 5 mL) was collected at 1st (6–15 weeks), 2nd (24–27 weeks) and 3rd (29–43 weeks) trimester from participants. Cord whole blood (about 2 mL) was sampled during delivery. In total, 144 maternal blood and 48 cord blood samples were taken. All blood samples were kept at room temperature for 30 min, and centrifuged at 4000× *g* for 10 min at 4 °C. The supernatant plasma was transferred into a tube and temporarily stored at hospitals at −20 °C. All whole blood samples were shipped to laboratory on dry ice and stored at −80 °C until they were assessed.

The procedure with regard to sample pretreatment and instrumental analysis was similar to that of the previous study. Briefly, the plasma samples were thawed and balanced to room temperature; 0.1 mL plasma sample was transferred into a 2 mL tube; 0.1 mL of mixed rhodium (Rh), indium (In), and rhenium (Re) were added as internal standard; and 1.8 mL of 1% nitric acid was added to acidified samples. The mixture was shaken and injected to instrument for identification and quantification of target analytes. The concentration of B, Al, Ni, Cr, Mn, As, Se, Sr, and Fe was determined by 7700x ICP-MS (Agilent Technologies, USA) and the remaining elements were determined by Elan DRC type II ICP-MS (The Perkin-Elmer Corporation, USA).

Results evaluated all took account of current thresholds and reference values for elements currently available.

### 2.3. Statistical Analysis

The basic characteristics of the participants contain number of participants, mean value, and the standard deviation for each group. Metal concentrations below the detection limit were imputed as 1/2 limit of detection (LOD). The median and interquartile range (IQR) were used to describe the distributions of the 18 metals. The Wilcoxon signed- rank test was used to compare the concentrations between maternal blood at 3rd trimester and cord blood, while the Friedman test was used to compare maternal blood during different pregnancy periods. The correlations between elements in cord plasma and 3rd trimester maternal plasma were assessed by Spearman correlation coefficients. All the statistical analyses were performed on the IBM SPSS Statistics 26.0 (IBM, Armonk, NY, USA). A two-sided *p*-value < 0.05 was considered significant.

## 3. Results

### 3.1. Population Characteristics

The demographic characteristics of participants in the study are shown in Table 1. In summary, 48 pregnant women were included in our cohort study, with average age 37.2 ± 1.9, birth weight 3226.0 ± 474.9 g, birth length 49.3 ± 2.3 cm.

Most women were Han (90%), while only 5 (10%) of them were from other ethnic groups. As to occupation, most women were categorised as worker/business/services (54%) or public official (32%), while the others were categorised as other (10%) or none (4%). Most women were undergraduates (52%) or graduate or higher (44%). Most participants did not have a scarred uterus (72%) and had a history of pregnancy and child bearing (52%).

### 3.2. Concentrations of Elements in Maternal Plasma during Pregnancy

**Figure 1:**
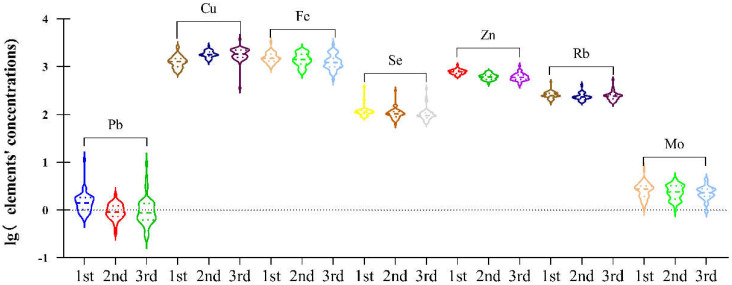
Violin plot of toxic and essential elements, in maternal plasma at 1st, 2nd and 3rd trimester. The plot was established based on the significant difference calculated by the Friedman test (*p* < 0.05) among plasma elements Pb, Cu, Fe, Se, Zn, Rb and Mo.

There was a significant difference in maternal plasma from 1st, 2nd to 3rd trimester, shown in Table 2 and Figure 1, with Pb (1.4 vs. 0.9 vs. 0.87 μg/L, *p* < 0.001), Se (111.3 vs. 102.65 vs. 95.28 μg/L, *p* < 0.001), Fe (1500.96 vs. 1413.25 vs. 1225.48 μg/L, *p* = 0.011), and Mo (2.75 vs. 2.4 vs. 2.31 μg/L, *p* = 0.043) decreasing during whole pregnancy. Concentrations of Cu (1272.96 vs. 1796.51 vs. 1844.87 μg/L, *p* < 0.001) increased during whole pregnancy. Notably, concentrations of Rb (248.26 vs. 230.95 vs. 242.01 μg/L, *p* = 0.002) displayed an initial decrease followed by a subsequent increase.

### 3.3. Concentrations of Elements in Cord Plasma and Maternal Plasma

**Figure 2:**
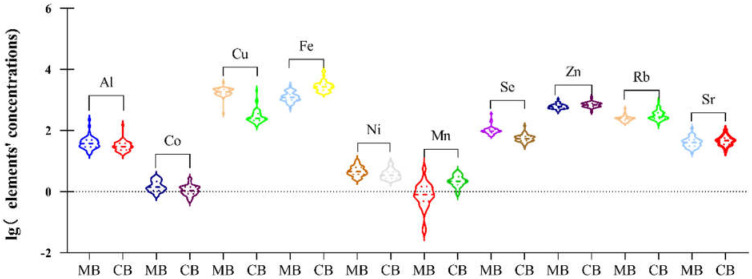
Violin plot of toxic and essential elements in maternal plasma at 3rd trimester and paired cord plasma. The plot was established based on the significant difference calculated by the Wilcoxon signed-rank Test (*p* < 0.05) among plasma elements Al, Co, Cu, Fe, Ni, Mn, Se, Zn, Rb, and Sr. MB means maternal blood and CB refers to cord blood.

As to concentrations of maternal plasma and cord plasma, shown in Table 3 and Figure 2, there was a significant difference in Al (37.33 vs. 29.51 μg/L, *p =* 0.004), Co (1.39 vs. 1.09 μg/L, *p* < 0.001), Cu (1844.87 vs. 249.98 μg/L, *p* < 0.001), Fe (1225.48 vs. 2139.78 μg/L, *p* < 0.001), Ni (4.51 vs. 3.41 μg/L, *p* = 0.007), Mn (0.79 vs. 2.17 μg/L, *p* < 0.001), Se (95.28 vs. 53.57 μg/L, *p* < 0.001), Zn (597.27 vs. 686.61 μg/L, *p* < 0.001), Rb (242.01 vs. 276.28 μg/L, *p* < 0.001), and Sr (40.07 vs. 46.58 μg/L, *p* = 0.021), with concentrations of Al, Co, Se, Cu, and Ni in cord plasma higher than in maternal plasma in 3rd trimester, while the rest were lower. Further information of placental transport efficiency (PTE) of 18 toxic and/or essential elements could be seen in the Supplementary Materials, Table S1.

In order to compare our results with previous literature, we review data on toxic and essential elements in maternal blood (MB) and cord blood (CB) of pregnant women reported in previous studies (Results were put in Supplementary Materials, Table S2).

### 3.4. Spearman Correlations between 18 Elements in Maternal Plasma at 3rd Trimester

**Figure 3:**
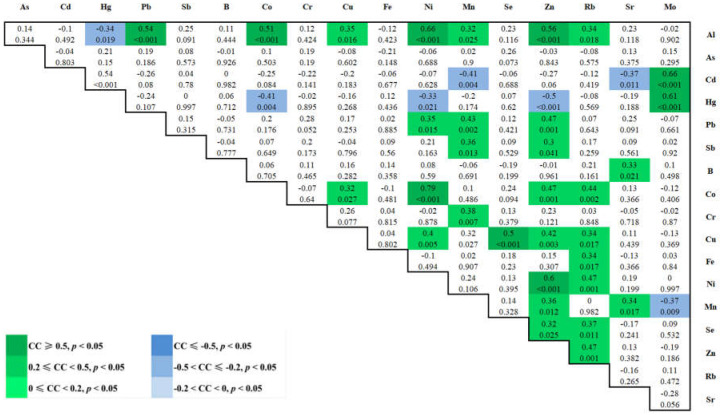
Color map of the Spearman correlations between 18 elements in maternal blood at 3rd trimester.

**Figure 4:**
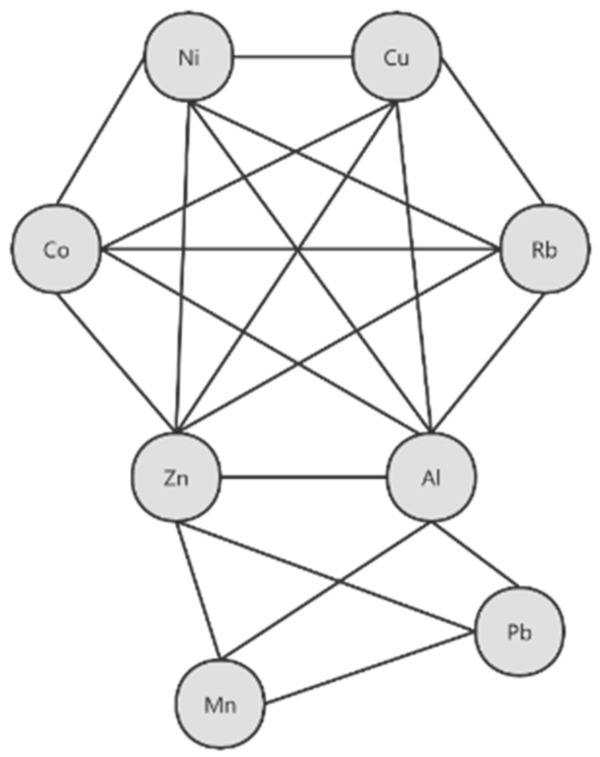
Toxic and essential trace elements’ correlation network in maternal plasma of pregnant women.

A total of 153 Spearman correlations between each two elements in maternal plasma with *p* values were obtained as shown in Figure 3. Interestingly, we found that elements Ni, Co, Cu, Al, Rb, and Zn all positively correlated with each other (*p* < 0.05) and formed a cluster, while Zn, Mn, Al, and Pb positively correlated with each other (*p* < 0.05), forming another cluster (Figure 4).

Correlation coefficients (CC, above) and *p* values (below) for the Spearman correlation between blood trace elements were calculated. The different intensities of correlation are represented by different colors.

The network was established based on the significant correlations (*p* < 0.05) among plasma elements Ni, Co, Cu, Al, Rb, Zn, Mn, and Pb.

### 3.5. Spearman Correlations between 18 Elements in Cord Plasma

**Figure 5:**
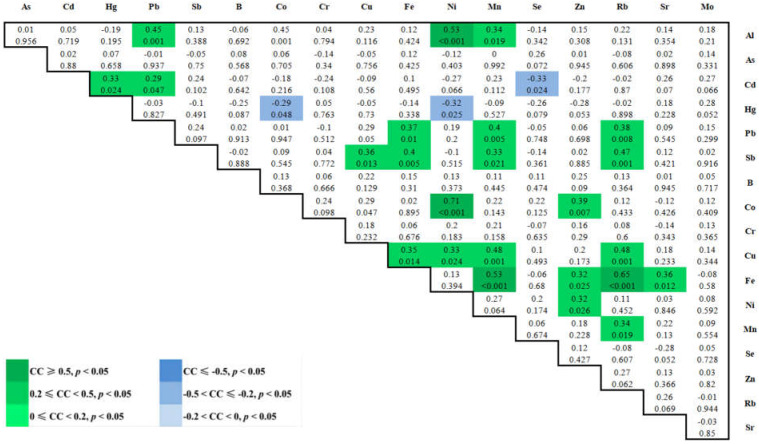
Color map of the Spearman correlations between 18 elements in cord plasma.

**Figure 6:**
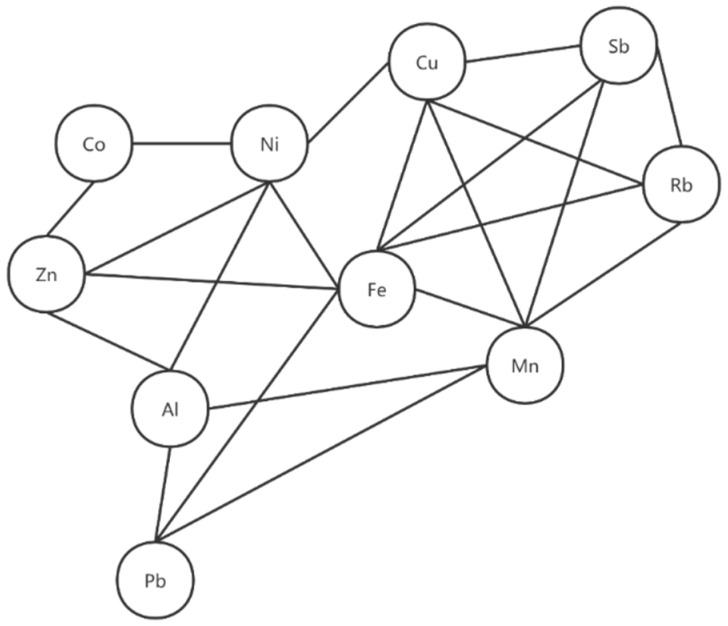
Toxic and essential trace elements’ correlation network in maternal plasma of pregnant women.

Similarly, a total of 153 Spearman correlations between each two elements in cord plasma with *p* values were obtained as shown in Figure 5. We found that Zn-Ni-Co, Zn-Ni-Fe, Mn-Al-Pb, Fe-Pb-Mn, Fe-Ni-Cu, and Rb-Cu-Sb-Fe-Mn formed positively- connected clusters (*p* < 0.05) (Figure 6).

Correlation coefficients (CC, above) and *p* values (below) for the Spearman correlation between blood trace elements were calculated. The different intensities of correlation are represented by different colors.

The network was established based on the significant correlations (*p* < 0.05) among plasma elements Co, Zn, Ni, Al, Pb, Fe, Cu, Mn, Rb, Sb.

### 3.6. Correlation Analysis of Trace Elements in Maternal Blood (MB) at 3rd Trimester and in Cord Blood (CB)

As to the correlation between concentrations in maternal plasma and cord plasma, we found that As (r = 0.383; *p* = 0.007), Sr (r = 0.444; *p* = 0.002) and Mo (r = 0.416; *p* = 0.003) have a significant correlation, as shown in Table 4.

## 4. Discussion

### 4.1. Summary of the Study

We found that concentrations of Pb, Se, Fe, Zn, and Mo all dropped during pregnancy, while Cu increased. Interestingly, concentrations of Rb decreased first but subsequently increased. In addition, elements such as Al, Co, Se, Cu, and Ni showed significantly lower levels in cord than in maternal plasma, while Sr, Fe, Rb, Mn, and Zn displayed significantly higher levels in cord than in maternal plasma.

Moreover, positively-interacted clusters were found in Ni-Co-Cu-Al-Rb-Zn and Zn-Mn-Al-Pb in maternal blood. Similar positively-interacted clusters were found in Zn-Ni-Co, Zn-Ni-Fe, Mn-Al-Pb, Fe-Pb-Mn, Fe-Ni-Cu, and Rb-Cu-Sb-Fe-Mn in cord plasma.

In addition, correlations between paired maternal and cord blood samples for As, Sr, and Mo were statistically significant, indicating that the fetus burden may reflect maternal exposure to some extent.

### 4.2. Concentration of Trace Elements during Pregnancy

We found that Pb, Se, Fe, Ni, Zn, and Mo levels investigated here decreased significantly during pregnancy, possibly because of fetus’ mobilization during pregnancy (Table 2, Figure 1). However, it should be stressed that the total blood volume increase varied from 20% to 100% above pre-pregnancy levels, usually close to 45% [17], and therefore the finding must consider the diluting effect of expansion of maternal plasma volume.

Pb exposure is a persistent global health hazard, with no safe exposure threshold [18]. Pb is a naturally occurring non-essential element, and human industrial practices can promote Pb exposures through the contamination of dust, food, and water [19]. Higher demand for calcium during pregnancy leads to increased bone turnover and increased circulating Pb levels [20]. Previous studies reported that the 3rd trimester is the period during pregnancy that contains the greatest mobilization of Pb from maternal bone and fastest fetal growth [21]. However, in our studies, the overall trend of Pb during pregnancy was downwards, likely due to larger plasma volume. Pb was detected in all cord blood samples, confirming its placental transfer. Cord blood lead has been used in many studies as an index of prenatal lead exposure and is considered as a potential predictor of child development [22]. Though we found no correlation between maternal and cord blood lead levels, it seems the placenta may still partially hinder the passage of lead to the fetus and reduce its toxic effect [23]. Notably, other studies reported significantly higher blood levels of Pb than in our study. In addition to differences in exogenous exposure, blood Pb concentrations can vary because of changes in hematocrit and Ca levels, plasma volume, and mobilization of Pb from bones during pregnancy [24,25].

It has been universally recognized that Se has many functions in the body, primarily as selenocysteine-containing proteins (seleno proteins). Se deficiencies can play an important role in adverse outcomes such as miscarriages, neural tube defects, diaphragmatic hernia, premature birth, low birth weight, pre-eclampsia, glucose intolerance, and gestational diabetes [26]. We found that Se content declined during pregnancy. Previous studies have found that Se stores in the body are depleted during the course of pregnancy, with most depletion occurring at the end of pregnancy [27,28].

Fe is an essential, multifunctional micronutrient. The ability of Fe to easily transition between two oxidation states (Fe^2+^ and ferric Fe^3+)^ underlies its involvement in a broad range of biological processes including oxygen transport, function of the electron transport chain, and DNA synthesis [29]. Fe supply to the fetus is wholly dependent on the transfer across the placenta. The flux of Fe through the placenta is unidirectional, and is greatest in the 3rd trimester, with several milligrams of Fe transferred to the fetus daily [30]. Considering that placental transfer of Fe is dependent on the bioavailability of Fe in the maternal circulation, the decrease in concentrations of Fe during whole pregnancy may arise from mobilization of the fetus and the diluting effect of maternal plasma volume.

Similar to Fe, a fall in Zn levels during pregnancy has been reported by many researchers and found also in our study [31,32,33,34,35,36]. The decrease in serum zinc concentration during pregnancy may reflect maternal–fetal Zn transfer in response to fetal growth [31]. Also, the expansion of maternal plasma volume can cause part of the dilution [33]. Furthermore, previous studies have suggested that the decrease in circulating Zn could be related to hemodilution, decreased levels of Zn-binding protein and hormonal changes [25]. The main characteristics associated with Zn deficiency include weight loss, failure to thrive, and enhanced susceptibility to infections; while Zn supplementation may have a positive effect on neonatal immune status and infant asthma from infectious diseases, as well as a reduced risk of preeclampsia in pregnant women and preterm births [37].

On the contrary, Cu levels significantly increase during pregnancy, probably due to mothers’ increased metabolic demand for these nutrients [25]. In fact, Cu is mobilized in the mother during pregnancy, resulting in a significant increase in maternal serum Cu concentrations compared to umbilical cord serum. It should be mentioned that Cu deficiency can lead to anemia, neutropenia, bone disease, and growth retardation in pediatric patients, as well as an increased risk of preterm births [25]. Furthermore, low Cu in early pregnancy is a risk factor for spontaneous abortion and CNS malformations, so supplementation before conception seems essential, and low Cu in later pregnancy is a risk factor for premature rupture of membrane [37].

Interestingly, we found that concentrations of Rb initially decreased but subsequently bounced back during pregnancy. Previous studies have reported that Rb exhibited negative associations with miscarriages [38]. To our knowledge, no previous research has reported similar results. We speculate that the initial decrease may arise from the rapid increase in plasma volume in early pregnancy [17], while the subsequent increase could be due to maternal mobilization in late pregnancy. More studies are needed to investigate this.

However, as maternal plasma volume significantly increases during pregnancy, those elements that concentrations remain stable during pregnancy undergo an actual increase of content. In particular, the variation patterns of essential trace elements including B, Cr, and Mo could be due to more maternal mobilization in late pregnancy, while results of toxic elements including As, Cd, and Hg were similar to previous studies [25,39,40]. More evidence is needed to explain this phenomenon.

### 4.3. Concentration of Trace Elements in Paired Maternal and Cord Plasma

The concentration of elements in the umbilical cord plasma of newborns influences the organism of the developing fetus and the adaptation of the newborn after birth to ectopic life, regulating several vital processes. Some elements are retained by the placental barrier, thus preventing them from entering the developing child’s body; however, the placenta is not an effective barrier for some xenobiotic elements as they are observed in the cord blood of newborns.

In our study, we mainly found lower levels of Al, Co, Se, Cu, and Ni in cord than in paired maternal plasma, which may arise from partial placental barrier, or less need on the part of the fetus [41] (Table 3, Figure 3).

The major routes of human exposure to Al include the respiratory tract, gastrointestinal tract, and skin [42]. Multiple epidemiological studies have reported an association between Al exposure and adverse pregnancy outcomes, including placental abruption [43], low birth weight [44], and birth defects [45]. Lower Al concentrations in cord blood may suggest that placenta partially blocks its transfer. To our knowledge, no previous study has investigated the association between Al concentrations in maternal serum and placental tissue.

Co, as an essential component of vitamin B12, is mainly acquired from dietary sources [46]. Previous studies have shown that lower maternal serum Co concentration might be associated with pregnancy-induced hypertension syndrome [47] and preterm births [48] in China’s population.

In this study, Cu content was comparatively lower in cord than in maternal plasma, similar to the results of other research [25,49,50], indicating a limited transplacental passage of Cu from mother to fetus. This might arise from low ceruloplasmin in the serum of newborns binding 96% of serum Cu [51]. In fact, ceruloplasmin could not penetrate the human placenta as the cord blood Cu did not strongly correlate with the maternal blood or colostrum concentrations, which is consistent with previous studies [52].

On the contrary, we found that Sr, Fe, Rb, Mn, and Zn demonstrated a higher level in cord than in paired maternal plasma, indicating that the placenta is no barrier for these elements, and that these elements may actively transport via placenta, consistent with previous studies [52] (Table 3, Figure 3).

Fe supply to the fetus is wholly dependent on transfer across the placenta. The flux of iron through the placenta is unidirectional, and is greatest in the 3rd trimester, with several milligrams of iron transferred to the fetus daily [30]. In this view, the mobilization of Fe might thus be the cause of the comparatively higher level of Fe in cord plasma.

Regarding the significant higher Mn levels in cord blood compared to maternal blood, it could reflect the active transport of this element from mother to fetus.

Mn is vital for the functioning of a healthy brain and nervous system, as well as maintaining metabolism and hormone production [37]. Mn levels appear to increase throughout pregnancy due to low iron levels and accelerated erythropoiesis associated with pregnancy [53]. The limited data available on placental Mn transfer suggest that Mn is transported actively since the Mn amount was significantly higher in umbilical cord blood than in maternal serum [52]. Reduced iron status in pregnancy and particularly late pregnancy may lead to increased uptake of dietary Mn due to an up-regulated iron absorption, since the intestinal transport mechanism for iron is unable to differentiate between iron and Mn [54]. Low levels of manganese are associated with lower birth weight [55] and possibly with preterm births [56]. However, since we did not find a correlation between paired maternal/cord blood Mn concentrations, other reasons may explain the higher Mn in cord blood, such as the lower or restricted elimination of Mn by the fetus or the inability of the fetus to utilize this element [57].

Notably, previous studies reported significantly higher levels of Mn than our study, which might result from different diets, use of supplements, or metals (as Fe) deficiency status, which might have something to do with age loss.

### 4.4. Correlation between Paired Samples of Trace Elements

We found a significant correlation of As, Sr and, Mo in paired maternal and cord plasma (Table 4).

Similar to our findings, significant correlations of As have been reported in the blood of mother/newborn pairs in South Africa, Belgium, Argentina and Spain [25,49,58], indicating that the developing fetus may be at risk of exposure to these elements via placental transfer. However, no previous studies have reported similar correlating relationships of Sr and Mo, to our knowledge.

Notably, correlation calculations for other paired trace elements investigated here show no significant direct or inverse correlations. It can be speculated that the missing correlations reflect that the uptake by nutrition, the body-pools and their mobilization of the mother during pregnancy are sufficiently high for an adequate supply of these elements to the fetus [51]. Other explanations are partial placental transfer and low demand from the fetus. 

### 4.5. Correlation between Two Element Concentrations

Positively-interacted clusters were found in Ni-Co-Cu-Al-Rb-Zn and Zn-Mn-Al-Pb in maternal blood, suggesting that those elements may arise from a similar source of exposure. In fact, exposure to metal mixtures rarely occurs alone, but more usually in the form of mixtures of common sources [59,60] (Figure 3 and Figure 4).

Similar positively-interacted clusters were found in Zn-Ni-Co, Mn-Al-Pb and Rb-Cu-Sb-Fe-Mn in cord plasma, suggesting that these elements may have similar placental transfer mechanisms or synergic interactions. (Figure 5 and Figure 6)

### 4.6. AMA Pregnancy

Since most toxic elements are cumulative and the burden rises with age, maternal age was thought to be associated with the concentrations of trace elements [58]. On the other hand, some essential elements see a reduction with age. Previous studies have reported age-dependent variations of Cu, Zn, Ca, Mg, Pb, Mn, As, Cd and Hg, with essential elements such as Cu, Ca, Mg, and Mn reducing over time while toxic elements such as Pb, As, Cd, and Hg increase [58].

However, it should be stressed that this study only featured pregnant women of AMA, without any comparison to younger pregnant women, and therefore the observed results are related to age and any transposition onto other age groups should be treated with caution.

## 5. Conclusions

We found that concentrations of Pb, Se, Fe, Zn, and Mo all dropped during pregnancy, while Cu went up. Interestingly, concentrations of Rb decreased initially but subsequently increased, which we cannot explain to our knowledge. Elements such as Al, Co, Se, Cu, and Ni showed significantly lower levels in cord than in maternal plasma, suggesting that the transplacental transfer of these nutrients was very limited. However, elements as Sr, Fe, Rb, Mn, and Zn displayed significantly higher levels in cord than in maternal plasma, suggesting that these elements may be essential to the growth of the fetus. Positively-interacted clusters were found in Ni-Co-Cu-Al-Rb-Zn and Zn-Mn-Al-Pb in maternal blood, suggesting that those elements may arise from a similar exposure source. Similar positively-interacted clusters were found in Zn-Ni-Co, Zn-Ni-Fe, Mn-Al-Pb, Fe-Pb-Mn, Fe-Ni-Cu, and Rb-Cu-Sb-Fe-Mn in cord plasma, suggesting that these elements could have similar transfer mechanisms or synergic effects. In addition, correlations between paired maternal and cord blood samples for As, Sr and Mo were statistically significant, indicating that the fetus burden may reflect the maternal exposure to some extent.

Although for most elements, the levels of toxic and essential elements in our cohort study were consistent with the scientific literature, it is well known and frequently reported that elements in the human body accumulate and are lost with age. Therefore, attention should be paid to exposure to trace elements in AMA. Furthermore, it should be noted that women with deficiencies in essential trace elements may show different dynamics during the course of pregnancy than those with sufficient levels. Further studies are needed to provide evidence of mechanisms of placental transport regarding these elements.

## Figures and Tables

**Table 1:** Demographic characteristics of participants in this study.

Variables	Mean ± SD/n (%)
Age (years)	37.2 ± 1.9
Birth weight (g)	3226.0 ± 474.9
Birth length (cm)	49.3 ± 2.3
Ethnic group	
Han	43 (89.6%)
Others	5 (10.4%)
Education level	
Secondary school	2 (4.2%)
Undergraduate	24 (50%)
Graduate or higher	22 (45.8%)
Occupation	
Worker/business/services	26 (54.2%)
Public official	15 (31.3%)
Others	5 (13.2%)
None	2 (5.3%)
Gravidity	
0	12 (25.0%)
≥1	36 (75.0%)
Parity	
0	24 (50.0%)
≥1	24 (50.0%)

SD: Standard deviation.

**Table 2:** Concentration of trace elements in maternal blood (MB) during pregnancy.

Elements	LOD	1st Trimester ^a^	2nd Trimester ^a^	3rd Trimester ^a^	*p* Value ^b^
Toxic elements					
Al	0.1	37.24 (31.02–46.38)	35.73 (32.8–43.01)	37.33 (28.83–48.8)	0.763
As	0.05	0.42 (0.22–0.74)	0.46 (0.28–0.81)	0.46 (0.25–1.04)	0.483
Cd	0.003	1.27 (0.98–1.44)	1.02 (0.64–1.23)	0.97 (0.65–1.29)	0.133
Hg	0.007	0.92 (0.49–1.19)	0.82 (0.3–1.09)	0.73 (0.27–1.22)	0.979
Pb	0.012	1.4 (1.03–1.82)	0.9 (0.74–1.21)	0.87 (0.63–1.37)	<0.001
Sb	0.003	3.78 (2.76–5.62)	3.47 (2.96–3.97)	3.69 (3.34–4.39)	0.920
Essential trace elements					
B	0.005	21.39 (16.52–26.84)	20.14 (16.16–23.22)	23.32 (16.04–29.67)	0.248
Co	0.001	1.41 (1.03–1.83)	1.41 (1.03–1.83)	1.39 (1.11–2.1)	0.717
Cr	0.002	0.61 (0.42–1.13)	0.68 (0.51–1.07)	0.66 (0.5–1.07)	0.524
Cu	0.03	1272.96 (1011.26–1477.93)	1796.51 (1666.11–2021.09)	1844.87 (1558.37–2219.37)	<0.001
Fe	0.9	1500.96 (1302.29–1759.41)	1413.25 (1154.64–1812.04)	1225.48 (989.72–1487.56)	0.011
Ni	0.005	4.26 (3.34–5.31)	4.02 (3.39–4.91)	4.51 (3.68–6.15)	0.305
Mn	0.008	0.66 (0.26–1.15)	0.95 (0.69–1.22)	0.79 (0.5–1.43)	0.281
Se	0.05	111.3 (104.12–120.88)	102.65 (89.58–114.11)	95.28 (86.14–108.22)	<0.001
Zn	0.06	781.89 (720.24–826.77)	612.57 (547.82–666.14)	597.27 (541.48–667.52)	<0.001
Rb	0.00396	248.26 (235.97–279.1)	230.95 (215.43–254.81)	242.01 (211.81–259.06)	0.002
Sr	0.01	34.54 (29.03–40.28)	33.37 (27.17–40.81)	40.07 (30.64–48.07)	0.071
Mo	0.002	2.75 (1.92–3.19)	2.4 (1.78–3.2)	2.31 (1.94–2.76)	0.043

^a^ Median (IQR). ^b^ *p* values were calculated by Friedman test to compare concentrations of elements in maternal plasma during different pregnancy periods. LOD: limit of detection. >LOD (%):total detectable rate of the three measurements.

**Table 3:** Differences in element concentrations in maternal plasma at 3rd trimester and cord plasma.

Elements	LOD	>LOD (%)	3rd Trimester ^a^	Delivery ^a^	*p* Value ^b^
Toxic elements					
Al	0.1	100%	37.33 (28.83–48.8)	29.51 (22.85–37.26)	0.004
As	0.05	91.7%	0.46 (0.25–1.04)	0.43 (0.26–0.76)	0.089
Cd	0.003	100%	0.97 (0.65–1.29)	0.93 (0.71–1.17)	0.806
Hg	0.007	97.9%	0.73 (0.27–1.22)	0.82 (0.63–1.13)	0.659
Pb	0.012	100%	0.87 (0.63–1.37)	0.78 (0.63–1.05)	0.412
Sb	0.003	100%	3.69 (3.34–4.39)	4.08 (3.08–4.63)	0.552
Essential trace elements					
B	0.005	100%	23.32 (16.04–29.67)	21.71 (15.35–27.67)	0.644
Co	0.001	100%	1.39 (1.11–2.1)	1.09 (0.85–1.41)	<0.001
Cr	0.002	100%	0.66 (0.5–1.07)	0.56 (0.35–0.94)	0.200
Cu	0.03	100%	1844.87 (1558.37–2219.37)	249.98 (214.09–351.26)	<0.001
Fe	0.9	100%	1225.48 (989.72–1487.56)	2663.86 (2139.78–3249.15)	<0.001
Ni	0.005	100%	4.51 (3.68–6.15)	3.41 (2.76–4.39)	0.007
Mn	0.008	91.7%	0.79 (0.5–1.43)	2.17 (1.74–3)	<0.001
Se	0.05	100%	95.28 (86.14–108.22)	53.57 (46.42–63.09)	<0.001
Zn	0.06	100%	597.27 (541.48–667.52)	686.61 (608.64–775.12)	<0.001
Rb	0.00396	100%	242.01 (211.81–259.06)	276.28 (246.35–378.05)	<0.001
Sr	0.01	100%	40.07 (30.64–48.07)	46.85 (35.24–56.22)	0.021
Mo	0.002	100%	2.31 (1.94–2.76)	2.14 (1.82–2.6)	0.126

^a^ Median (IQR). ^b^ *p* values were calculated by the Friedman test to compare concentrations of elements in maternal plasma during different pregnancy periods. LOD: limit of detection. >LOD (%): detectable rate of element in cord plasma.

**Table 4:** Spearman correlation analysis of trace elements in maternal blood (MB) at 3rd trimester and in cord blood (CB).

Elements	r	*p* Value ^a^
Toxic elements		
Al	0.023	0.878
As	0.383	0.007
Cd	0.184	0.211
Hg	−0.137	0.352
Pb	−0.042	0.777
Sb	−0.056	0.703
Essential trace elements		
B	0.176	0.231
Co	−0.014	0.926
Cr	−0.069	0.640
Cu	0.143	0.333
Fe	0.043	0.771
Ni	−0.087	0.557
Mn	−0.016	0.913
Se	0.194	0.187
Zn	0.171	0.245
Rb	0.263	0.071
Sr	0.444	0.002
Mo	0.416	0.003

^a^*p* value was calculated by Spearman correlation analysis.

## Data Availability

The datasets used and/or analyzed during the current study are available from the corresponding author on reasonable request.

## Supplementary Materials

The following supporting information can be downloaded at: https://www.mdpi.com/article/10.3390/ijerph192114485/s1, Table S1: Placental transport efficiency (PTE) of 18 toxic and/or essential elements; Table S2: Data on toxic and essential elements in maternal blood (MB) and cord blood (CB) of pregnant women reported in previous studies [1,2,3,10,25,49,50,58,60,61,62,63,64,65,66,67,68,69,70,71,72,73,74,75,76].

## Abbreviations

AMA	advanced maternal age
LOD	limit of detection
