# Supplementary material for: An Evaluation of Exposure to 18 Toxic and/or Essential Trace Elements Exposure in Maternal and Cord Plasma during Pregnancy at Advanced Maternal Age
Source: Int J Environ Res Public Health. 2022 Nov 4;19(21):14485. doi: 10.3390/ijerph192114485 (PMC9659256; doi:10.3390/ijerph192114485)
Supplement: Supplementary file 1 [file ijerph-19-14485-s001.zip › ijerph-1979646-supplementary.pdf]

Table S1. Placental transport efficiency (PTE) of 18 toxic and/or essential elements.*

Elements	Mean ± SD	Median
**Toxic elements**		
Al	94.1%±70.3%	82.5%
As	132.8%±147.1%	93.0%
Cd	120.3%±79.9%	98.4%
Hg	404.8%±838.6%	111.1%
Pb	130.4%±119.1%	82.0%
Sb	107.8%±43.0%	104.9%
**Essential trace elements**		
B	161.5%±365.0%	97.4%
Co	86.3%±58.9%	74.3%
Cr	105.9%±85.9%	86.0%
Cu	19.4%±17.8%	13.7%
Fe	253.2%±145.4%	207.3%
Ni	91.6%±55.0%	71.0%
Mn	575.6%±1058.1%	286.8%
Se	56.6%±16.3%	54.0%
Zn	116.7%±25.4%	116.0%
Rb	135.5%±50.4%	125.0%
Sr	123.2%±47.9%	108.2%
Mo	98.6%±35.9%	93.0%

*: Placental transport efficiency was measured by concentrations of cord plasma/ concentrations of maternal plasma at 3rd trimester of pregnancy. This parameter was initially a supplement to placental barrier's ability which is speculated by comparison between concentrations of maternal plasma at 3rd trimester and cord plasma. However, to be succinct and clear, we decided to remove it from our manuscript.

Table S2. Data on toxic and essential elements in maternal blood (MB) and cord blood (CB) of pregnant women reported in previous studies.

Elements	Country	Women n.	MB (µg/L)	CB (µg/L)	References
**Toxic elements**					
Al	South Africa	425		6.79	(Röllin et al. 2018)
	China	292	62.04		(Ma et al. 2022)
As	Canada	2001	0.69		(Ettinger et al. 2017)
	Flanders	235	0.64	0.54	(Baeyens et al. 2014)
	China	215	0.52		(Jin et al. 2014)
	South Africa	62	0.57	0.46	(Rudge et al. 2009)
	Australia	173	1.26		(Callan et al. 2013)
	North Norway	211	1.40		(Hansen et al. 2011)
	Japan	78			(Shirai et al. 2010)
	Italy	40	1.8		(Bocca et al. 2019)
	Italy	32		1.2	(Bocca et al. 2019)
Cd	Canada	2001	0.20		(Arbuckle et al. 2016)
	Flanders	235	0.31	0.07	(Baeyens et al. 2014)
	China	215	0.47		(Jin et al. 2014)
	China	209	0.48	0.15	(Sun et al. 2014)
	South Africa	62	0.15	0.02	(Rudge et al. 2009)
	Germany	50	0.34		(Kopp et al. 2012)
	South Korea	79	0.59	0.04	(Kim et al. 2015)
	North Norway	163	0.15		(Hansen et al. 2011)
	Saudi Arabia	1579	0.98	0.70	(Al-Saleh et al. 2014)
	Spain	140	0.60	0.27	(García-Esquinas et al. 2013)
	Japan	78			(Shirai et al. 2010)
	UK	4286	0.29		(Taylor et al. 2014)
	Italy	40	0.4		(Bocca et al. 2019)
	Italy	32		0.5	(Bocca et al. 2019)

Hg	Canada	2001	0.56	0.80	(Arbuckle et al. 2016)
	China	215	0.26		(Jin et al. 2014)
	China	292	0.28		(Ma et al. 2022)
	South Africa	62	0.65	1.2	(Rudge et al. 2009)
	Germany	50	0.44	1.48	(Kopp et al. 2012)
	South Korea	79	2.66	5.58	(Kim et al. 2015)
	North Norway	211	1.20		(Hansen et al. 2011)
	Saudi Arabia	1579	1.95	2.87	(Al-Saleh et al. 2014)
	Spain	140	4.61	7.66	(García-Esquinas et al. 2013)
	UK	4134	1.86		(Taylor et al. 2014)
	Italy	40	1.8		(Bocca et al. 2019)
	Italy	32		2.8	(Bocca et al. 2019)
Pb	Canada	2001	5.59	7.67	(Arbuckle et al. 2016)
	Flanders	235	11.1[a]	8.6[a]	(Baeyens et al. 2014)
	China	215	24.5		(Jin et al. 2014)
	China	209	40.5	32.3	(Sun et al. 2014)
	China	292	7.80		(Ma et al. 2022)
	Germany	50	11.5	10.3	(Kopp et al. 2012)
	South Korea	79	10.2	8.8	(Kim et al. 2015)
	North Norway	210	7.50		(Hansen et al. 2011)
	Saudi Arabia	1579	25.4	20.5	(Al-Saleh et al. 2014)
	Spain	140	18.9	13.8	(García-Esquinas et al. 2013)
	Japan	78			(Shirai et al. 2010)
	UK	4285	34.1		(Taylor et al. 2014)
	Italy	40	12		(Bocca et al. 2019)
	Italy	32		7.9	(Bocca et al. 2019)
**Essential elements**					
B	Israel	40	122		(Silberstein et al. 2015)
	Argentina	194	133		(Igra et al. 2016)

Co	South Africa	62	0.60	0.27	(Rudge et al. 2009)
	Western Australia	173	0.18		(Callan et al. 2013)
	China	292	0.19		(Ma et al. 2022)
	North Norway	211	0.10		(Hansen et al. 2011)
	Italy	40	0.3		(Bocca et al. 2019)
	Italy	32		0.3	(Bocca et al. 2019)
Cr	Australia	173	<1.0		(Callan et al. 2013)
	Ankara	100	0.337	0.121	(Yüksel et al. 2021)
	China	52	0.177	0.075	(Zhou et al. 2019)
	China	156	3.42	7.44	(Li et al. 2019)
	Italy	40	0.5		(Bocca et al. 2019)
	Italy	32		0.6	(Bocca et al. 2019)
Cu	Flanders	235	1312	600	(Baeyens et al. 2014)
	South Africa	62	1730	657	(Rudge et al. 2009)
	Germany	50	1120	470	(Kopp et al. 2012)
	Australia	173	1252		(Callan et al. 2013)
	America	1857	1874		(Zheng et al. 2019)
	North Norway	211	1650		(Hansen et al. 2011)
	Japan	78			(Shirai et al. 2010)
	China	292	1807.49		(Ma et al. 2022)
	Italy	40	1664		(Bocca et al. 2019)
	Italy	32		623	(Bocca et al. 2019)
Fe	Israel	40	1857		(Silberstein et al. 2015)
	China	52	624.02	536.35	(Zhou et al. 2019)
	Taiwan, China	145	292.30	414.40	(Huang et al. 2017)
Ni	Ankara	100	0.128	0.099	(Yüksel et al. 2021)
	China	156	12.8	4.47	(Li et al. 2019)
	China	292	1.50		(Ma et al. 2022)
	Australia	173	<2.0		(Callan et al. 2013)

	Italy	40	0.6		(Bocca et al. 2019)
	Italy	32		0.7	(Bocca et al. 2019)
Mn	Canada	2001	12.6	31.9	(Arbuckle et al. 2016)
	Flanders	235	12.1	31.2	(Baeyens et al. 2014)
	South Africa	62	16.8	34.9	(Rudge et al. 2009)
	South Africa	441	15.85		(Okereafor et al. 2020)
	Germany	50	17.0	28.8	(Kopp et al. 2012)
	Australia	173	9.14		(Callan et al. 2013)
	North Norway	211	10.7		(Hansen et al. 2011)
	Italy	40	16		(Bocca et al. 2019)
	Italy	32		28	(Bocca et al. 2019)
Se	South Africa	62	104	111	(Rudge et al. 2009)
	South Africa	444	62.82		(Okereafor et al. 2020)
	Australia	173	88		(Callan et al. 2013)
	America	1857	123		(Zheng et al. 2019)
	Norway	211	85		(Hansen et al. 2011)
	Japan	78			(Shirai et al. 2010)
	China	209	131	126	(Sun et al. 2014)
	China	292	88.03		(Ma et al. 2022)
	Italy	40	107		(Bocca et al. 2019)
	Italy	32		100	(Bocca et al. 2019)
Zn	South Africa	62	6290	2548	(Rudge et al. 2009)
	South Africa	446	497.8		(Okereafor et al. 2020)
	Germany	50	5120	1340	(Kopp et al. 2012)
	Australia	173	2330		(Callan et al. 2013)
	North Norway	211	5110		(Hansen et al. 2011)
	America	1857	806		(Zheng et al. 2019)
	Italy	40	6708		(Bocca et al. 2019)
	Italy	32		2311	(Bocca et al. 2019)

Sr	Israel	40	54	(Silberstein et al. 2015)
	China	292	26.5	(Ma et al. 2022)
Mo	America	1857	1.9	(Zheng et al. 2019)

References

Al-Saleh I, Shinwari N, Mashhour A, Rabah A. 2014. Birth outcome measures and maternal exposure to heavy metals (lead, cadmium and mercury) in saudi arabian population. Int J Hyg Environ Health 217:205-218.

Arbuckle TE, Liang CL, Morisset A-S, Fisher M, Weiler H, Cirtiu CM, et al. 2016. Maternal and fetal exposure to cadmium, lead, manganese and mercury: The mirec study. Chemosphere 163:270-282.

Baeyens W, Vrijens J, Gao Y, Croes K, Schoeters G, Den Hond E, et al. 2014. Trace metals in blood and urine of newborn/mother pairs, adolescents and adults of the flemish population (2007-2011). Int J Hyg Environ Health 217:878-890.

Bocca B, Ruggieri F, Pino A, Rovira J, Calamandrei G, Martínez MÁ, et al. 2019. Human biomonitoring to evaluate exposure to toxic and essential trace elements during pregnancy. Part a. Concentrations in maternal blood, urine and cord blood. Environ Res 177:108599.

Callan AC, Hinwood AL, Ramalingam M, Boyce M, Heyworth J, McCafferty P, et al. 2013. Maternal exposure to metals--concentrations and predictors of exposure. Environ Res 126:111-117.

Ettinger AS, Arbuckle TE, Fisher M, Liang CL, Davis K, Cirtiu C-M, et al. 2017. Arsenic levels among pregnant women and newborns in canada: Results from the maternal-infant research on environmental chemicals (mirec) cohort. Environ Res 153.

García-Esquinas E, Pérez-Gómez B, Fernández-Navarro P, Fernández MA, de Paz C, Pérez-Meixeira AM, et al. 2013. Lead, mercury and cadmium in umbilical cord blood and its association with parental epidemiological variables and birth factors. BMC Public Health 13:841.

Hansen S, Nieboer E, Sandanger TM, Wilsgaard T, Thomassen Y, Veyhe AS, et al. 2011. Changes in maternal blood concentrations of selected essential and toxic elements during and after pregnancy. J Environ Monit 13:2143-2152.

Huang S-H, Weng K-P, Lin C-C, Wang C-C, Lee CT-C, Ger L-P, et al. 2017. Maternal and umbilical cord blood levels of mercury, manganese, iron, and copper in southern taiwan: A cross-sectional study. J Chin Med Assoc 80:442-451.

Igra AM, Harari F, Lu Y, Casimiro E, Vahter M. 2016. Boron exposure through drinking water during pregnancy and birth size. Environ Int 95:54-60.

Jin L, Liu J, Ye B, Ren A. 2014. Concentrations of selected heavy metals in maternal blood and associated factors in rural areas in shanxi province, china. Environ Int 66:157-164.

Kim Y-M, Chung J-Y, An HS, Park SY, Kim B-G, Bae JW, et al. 2015. Biomonitoring of lead, cadmium, total mercury, and methylmercury levels in maternal blood and in umbilical cord blood at birth in south korea. Int J Environ Res Public Health 12:13482-13493.

Kopp RS, Kumbartski M, Harth V, Brüning T, Käfferlein HU. 2012. Partition of metals in the maternal/fetal unit and lead-associated decreases of fetal iron and manganese: An observational biomonitoring approach. Arch Toxicol 86:1571-1581.

Li A, Zhuang T, Shi J, Liang Y, Song M. 2019. Heavy metals in maternal and cord blood in beijing and their

efficiency of placental transfer. J Environ Sci (China) 80.

Ma J, Zhang H, Zheng T, Zhang W, Yang C, Yu L, et al. 2022. Exposure to metal mixtures and hypertensive disorders of pregnancy: A nested case-control study in china. Environ Pollut:119439.

Okereafor U, Makhatha M, Mekuto L, Uche-Okereafor N, Sebola T, Mavumengwana V. 2020. Toxic metal implications on agricultural soils, plants, animals, aquatic life and human health. Int J Environ Res Public Health 17.

Röllin HB, Nogueira C, Olutola B, Channa K, Odland JØ. 2018. Prenatal exposure to aluminum and status of selected essential trace elements in rural south african women at delivery. Int J Environ Res Public Health 15.

Rudge CV, Röllin HB, Nogueira CM, Thomassen Y, Rudge MC, Odland JØ. 2009. The placenta as a barrier for toxic and essential elements in paired maternal and cord blood samples of south african delivering women. J Environ Monit 11:1322-1330.

Shirai S, Suzuki Y, Yoshinaga J, Mizumoto Y. 2010. Maternal exposure to low-level heavy metals during pregnancy and birth size. J Environ Sci Health A Tox Hazard Subst Environ Eng 45:1468-1474.

Silberstein T, Saphier M, Mashiach Y, Paz-Tal O, Saphier O. 2015. Elements in maternal blood and amniotic fluid determined by icp-ms. J Matern Fetal Neona 28:88-92.

Sun H, Chen W, Wang D, Jin Y, Chen X, Xu Y. 2014. The effects of prenatal exposure to low-level cadmium, lead and selenium on birth outcomes. Chemosphere 108:33-39.

Taylor CM, Golding J, Emond AM. 2014. Lead, cadmium and mercury levels in pregnancy: The need for international consensus on levels of concern. J Dev Orig Health Dis 5:16-30.

Yüksel B, Arıca E, Söylemezoğlu T. 2021. Assessing reference levels of nickel and chromium in cord blood, maternal blood and placenta specimens from ankara, turkey. J Turk Ger Gynecol Assoc 22:187-195.

Zheng Y, Zhang C, Weisskopf M, Williams PL, Parsons PJ, Palmer CD, et al. 2019. A prospective study of early pregnancy essential metal(loid)s and glucose levels late in the second trimester. J Clin Endocrinol Metab 104:4295-4303.

Zhou C, Zhang R, Cai X, Xiao R, Yu H. 2019. Trace elements profiles of maternal blood, umbilical cord blood, and placenta in beijing, china. J Matern Fetal Neona 32:1755-1761.
